# In Vitro Azole and Amphotericin B Susceptibilities of *Malassezia furfur* from Bloodstream Infections Using E-Test and CLSI Broth Microdilution Methods

**DOI:** 10.3390/antibiotics9060361

**Published:** 2020-06-26

**Authors:** Wafa Rhimi, Chioma Inyang Aneke, Adriana Mosca, Domenico Otranto, Claudia Cafarchia

**Affiliations:** 1 Dipartimento di Medicina Veterinaria, Università degli Studi “Aldo Moro”, 70010 Bari, Italy; wafa.rhimi@uniba.it (W.R.); chioma.aneke@uniba.it (C.I.A.); domenico.otranto@uniba.it (D.O.); 2 Department of Veterinary Pathology and Microbiology, University of Nigeria, Nsukka 410001, Nigeria; 3 Dipartimento Interdisciplinare di Medicina, Università degli Studi di Bari, 70124 Bari, Italy; adriana.mosca@uniba.it; 4 Faculty of Veterinary Sciences, Bu-Ali Sina University, Hamedan 65174, Iran

**Keywords:** *Malassezia furfur*, azole, amphotericin B, antifungal susceptibility profile, E-test, CLSI broth microdilution

## Abstract

The number of reports of *Malassezia furfur* bloodstream infections is constantly increasing and there is a need for more simple antifungal susceptibility methods for their management. In this study, a total of 39 *M. furfur* isolates collected from hospitalized patients with fungemia were screened for antifungal susceptibility to azole and amphotericin B (AmB) using Clinical and Laboratory Standards Institute broth microdilution (CLSI BMD) and E-test in Sabouraud dextrose agar + 1% Tween80 (SDAt) and mDixon agar (DIX). Essential agreement (EA) and discrepancies between the two methods were evaluated after 48 h and 72 h reading times. Itraconazole (ITZ) and posaconazole (POS) displayed the lowest MIC values whereas fluconazole (FLZ) and AmB the highest, regardless of the methods and the reading time. The EA between BMD was >95% for FLZ and voriconazole (VOR) regardless of the media in the E-tests and reading time. The EA between BMD with E-test for AmB was >97% only when E-test in SDAt was used. The EA between BMD and E-test for ITZ and POS varied according to the media in E-test procedures and the reading time and was higher than 66.6% (POS) or 72% (ITZ) only when SABt was used. Substantial discrepancies for ITZ and POS were >5.1% regardless of the media and the reading time. This study suggests that the E-test in SABt represents an alternative method to CLSI BMD to evaluate the susceptibility of *M. furfur* to FLZ, VOR and AmB and not for ITZ and POS.

## 1. Introduction

*Malassezia furfur* is a lyphophilic commensal organism of human and animal skin that may become pathogenic under the influence of predisposing factors leading to cases of dermatitis in immunocompetent patients as well as bloodstream infections (BSI) in immunocompromised host [1,2,3]. Clinical manifestations induced by *M. furfur* include skin disorders in humans and animals and fungemia in humans [2,3]. Recently the number of human and animal skin infections by *Malassezia* spp. as well as the occurrence of *M. furfur* BSI increased. For the treatment of *Malassezia*-related infections, azoles and the polyene amphotericin B (AmB) are frequently employed both in humans and in animals. Topical antifungal agents (mainly azoles) are adequate for the management of localized skin lesions, while systemic itraconazole (ITZ) or fluconazole (FLZ) for severe skin diseases [3]. AmB is effective in the treatment of *Malassezia* systemic infections, both in preterm infants and adults ([3]. However, reports of treatment failure with terbinafine in *M. furfur* skin infection and FLZ or posaconazole (POS) in *M. furfur* fungemia are starting to appear [4,5,6,7,8,9,10,11]. Overall, the above picture led to suggest the occurrence of resistance phenomena in this yeast species, also in relation to the occurrence of the synergic effect of the combination of FLZ or voriconazole (VOR) with modulators or inhibitors of drug efflux pumps (i.e., haloperidol and promethazine) in *M. furfur* and *M. pachydermatis* strains [12]. Standardized methods for in vitro evaluation of antifungal susceptibility are lacking for these yeasts, thus resulting in variable susceptibility profiles to azoles among *Malassezia* spp. and in the lack of clinical breakpoints. Since 2000, different data were published on antifungal susceptibility profile of *M. furfur* by using Clinical and Laboratory Standards Institute broth microdilution (CLSI BMD) methods by modifying the media, time of incubation and inocula, thus resulting in a variable azoles susceptibility of this yeasts species [3,8,13,14,15,16,17,18,19,20,21,22]. In 2014, it was suggested that Sabouraud dextrose broth plus 1% Tween 80 might be an optimal broth medium for testing the azole susceptibility of *M. furfur* using the CLSI BMD protocol [8]. However, reference BMD is difficult to incorporate in many laboratories because it is relatively costly and laborious and requires the storage of panels of drugs in a frozen or dehydrated format. 

Due to the constant growth of cutaneous or systemic *M. furfur* infections, there is a need for an alternative, easier and less expensive susceptibility testing method than CLSI BMD for managing *M. furfur* infections, mainly fungemia. Data about the usefulness of agar-based methods (i.e., disk diffusion (DD) and E-test) for testing the antifungal susceptibility of *Malassezia* spp. yeasts are scant and mainly focused on *M. pachydermatis* [5,21,22,23,24,25,26,27]. 

The lack of data about E-test procedures for testing the antifungal susceptibility of *M. furfur* strains coming from patients with fungemia spurred the interest in this study with the multiple aims to: (i) evaluate the antifungal susceptibility of *M. furfur* strains recovered from hospitalized patients with fungemia to FLZ, itraconazole (ITZ), VOR, POS and AmB using a modified CLSI broth microdilution (BMD) and the E-test in lipid media such as Sabouraud dextrose agar +1% Tween80 (SDAt) and mDixon agar (DIX) and (ii)) estimate the agreement of results obtained with the E-test procedures and CLSI BMD. Agreement of results was also evaluated at 48 h vs. 72 h reading times. 

## 2. Results

The results in repeated experiments obtained with CLSI (i.e., two duplicates) and E-test (i.e., two duplicates) were the same or ±1 log2 dilution from the initial results. All quality control MIC values determined in each antifungal test were within the ranges established by the CLSI (2008). In particular the following MIC values of *C. krusei* (FLZ = 64 µg/mL, ITZ = 0.5 µg/mL, VOR = 0.12 µg/mL, POS = 0.5 µg/mL, AmB = 2 µg/mL) and *C. parapsilosis* (FLZ = 8 µg/mL, ITZ = 0.32 µg/mL, VOR = 0.03 µg/mL, POS = 0.25 µg/mL, AmB = 1 µg/mL) were registered with CLSI BMD procedures.” Table 1 and Table 2 summarize the in vitro susceptibilities of *M. furfur* to FLZ, ITZ, VOR, POS and AmB obtained with different methods after 48 h and 72 h of incubation, respectively.

The EA and discrepancies relative to the antifungal drugs tested between methods were also reported. ITZ and POS displayed the lowest MIC values whereas FLZ and AmB the highest, regardless of the methods and the reading time (Table 1 and Table 2). The MIC values of azoles and AmB were higher when tested with E-test in DIX or SDAt than those registered by using CLSI BMD (Table 1 and Table 2). In addition, E-test MICs of POS and AMB using DIX were significantly higher than those using SDAt (Table 1 and Table 2).

The EA between BMD with E-test was > 95% for FLZ and VOR regardless of the media in the E-tests and the reading time (Table 1 and Table 2). The EA between BMD with E-test for AmB was >97% only when SABt was used in E-test (Table 1 and Table 2). The EA between BMD and E-test for ITZ and POS varied according to the media in E-test and the reading time and was higher than 66.6% (POS) or 72% (ITZ) only when SABt was used. “Nonsubstantial” or “substantial” discrepancies were registered for ITZ and POS using SDAt and DIX after 48 and 72 h of incubation (Table 1 and Table 2). Substantial discrepancies (5.1% for POS and 12.8% for ITZ) registered using SDAt did not vary after 48 and 72 h of incubation. Substantial discrepancies for POS at 48 h and/or 72 h in DIX (i.e., 40%) were higher than that registered in SDAt (i.e., 5.1%; Table 1 and Table 2).

## 3. Discussion

Data indicate that the E-test is a reliable method for evaluating the susceptibility of *M. furfur* to FLZ, AmB and VOR and not for ITZ and POS. The low susceptibility of *M. furfur* to FLZ and AmB by using the BMD method is confirmed by E-test procedures herein presented with an overall EA ≥ 97.4% when SDAt was used [8,13,14,15,16,17,18,19,20,21,28]. In particular, while both media (DIX and SABt) were useful to test the susceptibility of FLZ (EA = 100%), only SABt for AmB (EA = 97.4%). Accordingly, NDS were observed between the two methods and they were very low only when SDAt was employed. Considering that the same media should be used to achieve concordant BMD and E-test results [15], data herein reported suggest that the E-test procedures should be performed in SDAt to test the antifungal susceptibility of *M. furfur* to FLZ and AmB. Though FLZ and AmB values of MIC were slightly higher after 72 h than 48 h of incubation, the absence of any statistical differences in MIC values at these two time points indicates that the E-test procedures for *M. furfur* should be performed after 48 h of incubation. The above results were also confirmed by the finding that the EA between BMD and E-tests for FLZ and AmB did not vary accordingly with the reading time. Therefore, reading results after 48 h incubation might expedite the decision about drugs to be used in clinical cases of *M. furfur* BSIs, as assessed for *M. pachydermatis* [24]. Even if the low FLZ susceptibility of *M. furfur* is concordant with the negative outcome of BSI in human patients [6,8,9,10,11], the low in vitro efficacy of AmB registered herein as well as in previous studies [15,19], needs further confirmations. Indeed, a good clinical outcome has been registered by treating BSI neonatal and adult patients with AmB [3,8]. The synergic effect of AmB with other drugs during treatment needs to be further investigated [14]. While the high EA (>94%) between the E-test and the BMD reference procedure registered for VOR, indicates that both media are useful to perform the test, the reading time of 72 h should be preferred (i.e., EA higher at 72 h than 48 h; 97.5 vs. 94.9). Prolonged incubation also reduced the percentage of isolates showing NSD. 

Although the high activity of ITZ and POS against *M. furfur* was confirmed by using E-test procedures, the low EA between BMD and E-tests suggests the inability of the E-test in evaluating *M. furfur* susceptibilities to these drugs, in contrast with data obtained with *M. pachydermatis* [24]. The high hydrophobicity of *M. furfur* induced by the lipid-rich cell wall as well as the differences in the pathogenic mechanisms (i.e., biofilm formation) compared to *M. pachydermatis* might explain the difference between *M. furfur* and *M. pachydermatis* in terms of their susceptibilities to the most relevant drugs used as therapeutic drugs [2,3]. Interestingly, SD higher than 5% indicated that the E-test procedure was ineffective for in vitro testing the susceptibility of *M. furfur* to ITZ and POS [29,30].

In this study, the MIC values of POS and ITZ in E-test procedures were higher than those registered in BMD method, and cannot be explained by the trailing phenomena in BMD [15].

The better growth of *M. furfur* in agar media with lipid in E-test procedures than in BMD, might explain the over interpretation of the MICs. To our knowledge, a limited number of studies have compared the E-test and CLSI BMD methods for in vitro antifungal susceptibility of *M. furfur* and only one reported concordance between the E-test and BMD [15]. On the contrary, the results from other studies also by considering *M. pachydermatis* emphasized the need for standardization of the tests by multicenter laboratory studies [27,31,32].

Since studies on *M. furfur* from BSI are scant, a multicenter laboratory study has never been performed, but this limitation might be mitigated by the fact that MIC values were determined by two independent experiments and evaluated by three different operators, as previously reported [33].

In this study, a very good agreement between the E-test and BMD methods in susceptibility tests of *M. furfur* for AmB, FLZ and VOR was revealed by using SDAt as medium, an inoculum suspension equivalent to 1–5 × 10^6^ CFU/mL and 48 h and 72 h of incubation. The statistical analysis of the results shows that limits of agreement were small enough to confirm that the E-test method in SDAt can be used in place of the CLSI BMD for clinical purposes to test the AmB, FLZ and VOR susceptibilities of *M furfur* from BSI patients. The E-test method is simple to perform, it does not require any specialized laboratory equipment and can be used for the routine testing of this species.

## 4. Materials and Methods 

### 4.1. Malassezia Furfur Strains

A total of 39 *M. furfur* strains from the BSIs of 9 neonates were identified phenotypically (i.e., macroscopic, and microscopic morphology) and physiologically, as reported elsewhere [4,34,35]. Validation of the strains to species level was achieved by matrix-assisted laser desorption ionization-time of flight MS [36]. The strains were isolated from blood samples through central venous catheters (*n*. 9) and from various fluids (i.e., peritoneal and bronchoalveolar fluids -*n*. 4), secretes (urine *n*. 4) as well form skin (*n*. 26) of the infected patients. All *M. furfur* strains were deposited and stored at −80 °C in the fungal collection at the Department of Veterinary Medicine of the University of Bari (Italy) with the following code numbers: CD1030, CD1036–1039, CD1045, CD1072, CD1473–CD1485, CD1488–CD1495, CD1509–CD1510 and CD1515–CD1523. Prior to testing, each strain was subcultured at least twice onto DIX plates without antimicrobial agents at 32 °C for 72 h to ensure strain purity and viability.

### 4.2. Antifungal Susceptibility Tests

The in vitro susceptibility of *M. furfur* strains to antifungal compounds was assessed employing the reference CLSI M27-A2 method, using the Sabouraud dextrose broth (Liofilchem Diagnostici, Roseto degli Abruzzi, Italy) with 1% of Tween 80 (Sigma-Aldrich Milan, Italy) as previously reported [8]. The following antifungal drugs were supplied by the manufacturers as pure standard compounds: AmB and ITZ (Sigma-Aldrich Milan, Italy), FLZ and VOR (Pfizer Pharmaceuticals; Groton, Connecticut, USA) and POS (Schering-Plough Corporation, Kenilworth, New Jersey, USA). FLZ was dissolved in sterile water, whereas the remaining drugs were solubilized in DMSO (Sigma-Aldrich Milan, Italy). The concentration of each antifungal drug ranged from 0.008 to 16 μg/mL, except for FLZ (i.e., from 0.06 to 128 μg/mL). Visual reading of plates was performed after 48 and 72 h of incubation at 32 °C. For azoles, the MIC endpoint was defined as the lowest concentration that produced a prominent decrease in turbidity (50%) relative to that of the drug-free control. The MIC for AmB was defined as the lowest concentration at which no visible growth could be detected. The E-test procedure was performed on the SDAt and on DIX. For the E-test procedure in DIX, 20 representative *M. furfur* strains were selected on the basis of their origin preferring strains from blood and body fluids (i.e., *n*. 6 from blood samples, *n*. 4 from various fluids, *n*. 4 from urine and *n*. 6 from the skin of the infected patients). The E-test strips (AB BIODISK, Solna, Sweden) for ITZ, VOR, POS and AmB (concentrations ranging from 0.002–32 mg/L) and for FLZ (from 0.16–256 mg/L) were used in the testing. Stock inoculum suspensions were prepared from 5 days old colonies grown on DIX, suspended in 5 mL sterile distilled water and thoroughly vortexed to achieve a smooth suspension. Turbidity was adjusted to an optical density of 2.4 using a turbidimeter (DEN-1 McFarland Densitometer, Biosan) which was equivalent to 1–5 × 10^6^ colony-forming unit (CFU)/mL, as validated by quantitative plate counts of CFU on DIX and SDAt. Sterile cotton swabs were dipped into the inoculum suspension and then streaked onto SDAt and DIX. The inoculated plates were left to stand at room temperature for 15 min, or until the excess moisture was completely absorbed, before applying E-test strips. The plates were incubated at 32 °C and read at 48 h and 72 h. The MIC endpoint was the drug concentration that resulted in complete inhibition of growth, including all microcolonies, hazes and isolated colonies. Two different tests for each isolate were performed with different methods. When the results were not concordant, the E-test and CLSI procedures were repeated and the results in agreement with previous initial results were considered for analysis. All the BMD and E-tests results were evaluated by three different operators independently. Data obtained were reported as MIC ranges, MIC mean value (MICm) and MIC at which 50% (MIC_50_) and 90% (MIC_90_) of the isolates were inhibited. Quality control strains (*Candida parapsilosis* ATCC 22019 and *Candida krusei* ATCC 6258 from the American Type Culture Collection, Manassas, VA, USA) were included to assess the accuracy of the drug dilutions and the reproducibility of the results [37].

### 4.3. Data Analysis

Both on-scale and off-scale results were included in the analysis. The low and high off-scale MICs were converted as the lowest MIC or the highest MIC, respectively. Essential agreement between the MIC values determined by the BMD and the E-test procedures was defined as a discrepancy of no more than two 2-fold dilutions [38]. In the absence of clinically validated MIC breakpoints for antifungal susceptibility testing of *M. furfur*, discrepancies were classified as nonsubstantial (NSD = discrepancies in MIC results of 3 or 4 2-fold dilutions) or substantial (SD = discrepancies of > 4 2-fold dilutions) [27,38]. The mean MIC values obtained using the same drugs in different test procedures were statistically analyzed using one-way Analysis of variance (ANOVA). The Chi-square test (χ2) to compare the discrepancies observed by using E-test in the two different media and CLSI BMD. A value of *p* < 0.05 was considered statistically significant.

## 5. Conclusions

In conclusion, this study confirms that the *M. furfur* from BSIs were highly susceptible to POS and ITZ, and less to FLZ and AmB [8,15,18,20,29]. The very low in vitro susceptibility of AmB needs to be confirmed and the clinical relevance of these in vitro results require further investigation. The good agreement between the E-test performed using SABt and the reference BMD method provides confirmation of the reliability of the former method to test the susceptibility of *M. furfur* to FLZ, VOR and AmB and not for ITZ and POS

## Figures and Tables

**Table 1:** Fluconazole (FLZ), itraconazole (ITZ), voriconazole (VOR), posaconazole (POS) and amphotericin B (AmB) minimum inhibitory concentration (MIC; μg/mL) data after 48 h of incubation obtained with Clinical and Laboratory Standards Institute broth microdilution (CLSI BMD) and E-test in Sabouraud + 1% Tween 80 and in mDixon of *Malassezia furfur* strains from immunocompromised patients with fungemia. Essential agreement (EA) and discrepancies among MIC obtained by E-tests compared with the CLSI BMD method were also reported.

Drugs	Antifungal Test	MIC Range	MIC_50_	MIC_90_	mMIC (sd)	EA with CLSI BMD	Discrepancies	
							NSD	SD
							N/Tot (%)	N/Tot (%)
FLZ	CLSI BMD	32–128	128	128	91.2 (40.7) ^a^			
	E-test SAB + Tween	32–128	64	128	89.2 (34.5) ^d,f^	100	0/39 (0)	0/39 (0)
	E-test mDixon	32–256	96	96	92.4 (68.7) ^i^	100	0/20 (0)	0/39 (0)
ITZ	CLSI BMD	0.008–4	0.06	0.25	0.2 (0.6) ^a,b^			
	E-test SAB + Tween	0.064–4	0.19	0.5	0.5 (0.8) ^d^	69.2	7/39 (17.9)	5/39 (12.8)
	E-test mDixon	0.38–1.5	0.75	1	0.8 (0.2) ^i,j^	60	6/20 (30)	2/20 (10)
VOR	CLSI BMD	0.03–4	1	1	0.8 (0.6) ^a^			
	E-test SAB + Tween	0.32–4	0.75	2	1.1 (0.8) ^d,f,g^	94.9	1/39 (2.6)	1/39
	E-test mDixon	0.125–3	0.5	3	1.1 (1) ^i,k^	100	0/20 (0)	0/20 (0)
POS	CLSI BMD	0.016–2	0.06	0.25	0.16 (0.3) ^a,c^			
	E-test SAB + Tween	0.19–1	0.125	0.1	0.4 (0.3) ^d,g,h^*	66.6	11/39 (28.2)	2/39 (5.1) *
	E-test mDixon	0.25–1.5	1.5	1.5	1.2 (0.5) ^i,j,l^*	40	4/20 (20)	8/20 (40) *
AmB	CLSI BMD	2–16	16	16	10.4 (6.5) ^a,b,c^			
	E-test SAB + Tween	4–32	32	32	24.5 (12.8) ^d,f,g,h^	97.4	1/39 (2.5) *	0/39 (0)
	E-test mDixon	32	32	32	32 (0) ^i,j,k,l^	75	5/20 (2.5) *	0/20 (0)

Nonsubstantial discrepancies (NSD = MIC results of 3 or 4 2-fold dilutions) or substantial discrepancies (SD = MIC results of > 4 2-fold dilutions; Statistically significant differences (*p* < 0.05) were reported with superscript asterisk and letters. Letters indicate statistical differences in mMIC values among drugs within the same testing procedure and asterisk indicate the statistical differences among mMIC values of each drug with different testing procedures and statistical differences between NSD and SD for each drug.

**Table 2:** Fluconazole (FLZ), itraconazole (ITZ), voriconazole (VOR), posaconazole (POS) and amphotericin B (AmB) minimum inhibitory concentration (MIC; μg/mL) data after 72 h of incubation obtained with CLSI BMD and E-test in Sabouraud + 1%tween 80 and E-test IN Dixon agar of *Malassezia furfur* strains from immunocompromised patients with fungemia. Essential agreements (EA) among MIC obtained by E-test compared with CLSI BD methods and discrepancies were also reported.

Drugs	Antifungal Test	MIC Range	MIC_50_	MIC_90_	mMIC (sd)	EA with CLSI BMD	Discrepancies	
							NSD	SD
							N/Tot (%)	N/Tot (%)
FLZ	CLSI BMD	32–128	128	128	111.2 (29.9) ^a^			
	E-test SAB + Tween	32–256	128	256	172.8 (84.4) ^e^	100	0/39 (0)	0/39 (0)
	E-test mDixon	64–256	96	128	110 (60.1) ^j^	100	0/20 (0)	0/39 (0)
ITZ	CLSI BMD	0.03–4	0.06	0.25	0.2 (0.2) ^a,b^			
	E-test SAB + Tween	0.064–4	0.19	0.5	0.5 (0.1) ^e,fn^	72	6/39 (15.4) *	5/39 (12.8)
	E-test mDixon	0.38–1.5	1	1.5	1.1 (0.3) ^j,k^*	40	8/20 (40) *	4/20 (20)
VOR	CLSI BMD	0.03–4	1	1	0.9 (0.6) ^a,b,c^			
	E-test SAB + Tween	0.32–4	0.75	2	1.1 (0.8) ^e,f,h^	97.4	0/39 (0)	1/39 (2.6)
	E-test mDixon	0.25–3	1.5	3	1.1 (1) ^j,l^	100	0/20 (0)	0/20 (0)
POS	CLSI BMD	0.016–2	0.06	0.25	0.16 (0.3) ^a,c,d^			
	E-test SAB + Tween	0.19–1	0.125	1	0.4 (0.3) ^e,h,i^*	66.6	11/39(28.2) *	2/39 (5.1) *
	E-test mDixon	0.5–1.5	1.5	1.5	1.3 (0.4) ^j,m^*	30	6/20 (30) *	8/20 (40) *
AmB	CLSI BMD	4–16	16	16	10.8 (5.9) ^a,b,c,d^			
	E-test SAB + Tween	6–32	32	32	24.5 (12.8) ^e,f,h,i^*	97.4	1/39 (2.5) *	0/39 (0)
	E-test mDixon	32	32	32	32 (0) ^j,k,l,m^*	75	5/20 (25) *	0/20 (0)

Nonsubstantial discrepancies (NSD = MIC results of 3 or 4 2-fold dilutions) or substantial discrepancies (SD = MIC results of > 4 2-fold dilutions; Statistically significant differences (*p* < 0.05) were reported with superscript asterisk and letters. Letters indicate statistical differences in mMIC values among drugs within the same testing procedure and asterisk indicate the statistical differences among mMIC values of each drug with different testing procedures and statistical differences between NSD and SD for each drug.
